# Change to a healthy diet in people over 70 years old: the PREDIMED experience

**DOI:** 10.1007/s00394-021-02741-7

**Published:** 2021-11-28

**Authors:** Rosa Casas, Margarida Ribó-Coll, Emilio Ros, Montserrat Fitó, Rosa-María Lamuela-Raventos, Jordi Salas-Salvadó, Itziar Zazpe, Miguel-Angel Martínez-González, Jose V. Sorlí, Ramon Estruch, Emilio Sacanella

**Affiliations:** 1 Department of Internal Medicine, Hospital Clinic, Institut d’Investigació Biomèdica August Pi i Sunyer (IDIBAPS), University of Barcelona, 170 Villarroel, 08036 Barcelona, Spain; 2 Ciber Fisiopatología de la Obesidad y la Nutrición (CIBEROBN), Instituto de Salud Carlos III, Madrid, Spain; 3 Lipid Clinic, Service of Endocrinology and Nutrition, IDIBAPS, Hospital Clinic, Barcelona, Spain; 4 Cardiovascular Risk and Nutrition and REGICOR Research Group, Hospital del Mar Medical Research Institute (IMIM), Barcelona, Spain; 5 Department of Nutrition and Food Science School of Pharmacy, University of Barcelona, Barcelona, Spain; 6 Human Nutrition Unit, Hospital Universitari de Sant Joan de Reus, IISPV, Universitat Rovira i Virgili, Reus, Spain; 7 Department of Preventive Medicine and Public Health, School of Medicine, University of Navarra, Pamplona, Spain; 8 Department of Epidemiology and Department of Biochemistry and Molecular Biology, School of Pharmacy and Nutrition, University of Navarra, Pamplona, Spain; 9 Department of Preventive Medicine and Public Health, School of Medicine, University of Valencia, Valencia, Spain

**Keywords:** Dietary habits, Cardiovascular risk factor, Mediterranean diet, Cardiovascular disease, Healthy diet, Fragility

## Abstract

**Purpose:**

It is difficult to change dietary habits and maintain them in the long run, particularly in elderly people. We aimed to assess whether adherence to the Mediterranean diet (MedDiet) and cardiovascular risk factor were similar in the middle-aged and oldest participants in the PREDIMED study.

**Methods:**

We analyzed participants belonging to the first and fourth quartiles of age (Q1 and Q4, respectively) to compare between-group differences in adherence to the nutritional intervention and cardiovascular risk factor (CRF) control during a 3-year follow-up. All participants underwent yearly clinical, nutritional, and laboratory assessments during the following.

**Results:**

A total of 2278 patients were included (1091 and 1187 in Q1 and Q4, respectively). At baseline, mean ages were 59.6 ± 2.1 years in Q1 and 74.2 ± 2.6 years in Q4. In Q4, there were more women, greater prevalence of hypertension and diabetes, and lower obesity and smoking rates than the younger cohort (*P* ≤ 0.001, all). Adherence to the MedDiet was similar in Q1 and Q4 at baseline (mean 8.7 of 14 points for both) and improved significantly (*P* < 0.01) and to a similar extent (mean 10.2 and 10.0 points, respectively) during follow-up. Systolic blood pressure, low density–lipoprotein cholesterol, and body weight were similarly reduced at 3 years in Q1 and Q4 participants.

**Conclusion:**

The youngest and oldest participants showed improved dietary habits and CRFs to a similar extent after 3 years’ intervention. Therefore, it is never too late to improve dietary habits and ameliorate CRF in high-risk individuals, even those of advanced age.

**Registration:**

The trial is registered in the London-based Current Controlled Trials Registry (ISRCTN number 35739639).

**Supplementary Information:**

The online version contains supplementary material available at 10.1007/s00394-021-02741-7.

## Introduction

Cardiovascular disease (CVD) has the highest incidence and prevalence in elderly persons, in whom it is associated with high morbidity and mortality [1, 2]. In Europe, CVD incidence is predicted to increase in the near future because of population ageing [3, 4]. In addition, the elderly frequently suffer multimorbidities, and a significant proportion can be classified as frail [5]. Frailty shares some physiopathological mechanisms with CVD and both conditions are tightly interrelated, as frailty can worsen CVD and CVD may precipitate frailty [6, 7]. A healthy dietary pattern, such as the Mediterranean diet (MedDiet) is associated with both lower rates of non-communicable diseases, including CVD [8, 9] and frailty [10, 11].

CVD is a main component of multimorbidity, and may be prevented and managed by pharmacological and non-pharmacologic therapies, such as a healthy diet, increased physical activity, and abstention from smoking [12]. In fact, lifestyle changes are the cornerstone of CVD prevention and are recommended in all international guidelines [12, 13]. The main problem with lifestyle measures is that a change in patient behaviour is mandatory to transform unhealthy habits to healthy ones and maintain them in the long run, which is particularly difficult in the elderly, due to several factors such as lower appetite, food changes, declining physical function, cooking for one, shopping for one, food cost, etc. [14–17].

The PREvención con DIeta MEDiterránea (PREDIMED) randomized trial demonstrated that a MedDiet supplemented with extra-virgin olive oil (EVOO) or mixed nuts can improve cardiovascular risk factor (CRF) control [18, 19] and reduce CVD incidence by nearly 30% in older individuals at high cardiovascular risk compared to advice on a low-fat diet (LFD) [9]. However, it is unknown whether adherence to the MedDiet was similar in the youngest and oldest participants in the PREDIMED trial and also whether the beneficial effect on CRFs differed in younger and older individuals, since in a previous PREDIMED study, short and long-term predictors of dietary changes were analyzed, but age was not a clear predictor in either of these two analyses [17].

## Materials and methods

### Study participants and design

The PREDIMED study (www.predimed.es) was a nutritional intervention–based randomized trial, single-blind, multicenter (11 centres throughout Spain) study carried out on 7447 participants at high vascular risk, but no CVD at enrolment, conducted from October 2003 to December 2010. The aim of this primary prevention study was to assess the long-term effects of the MedDiet on incident CVD in men and women aged 55–80 years at high CVD risk. The protocol, methods, design, and eligibility criteria for this study have been reported in detail elsewhere [9]. Briefly, participants were assigned to one of three nutritional interventions by a computer-generated random-number sequence: a traditional MedDiet supplemented with either complementary EVOO or tree nuts and a control diet based on advice to follow an LFD.

In the current study, we analyzed data of participants belonging to the first (*n* = 1091) and fourth (*n* = 1187) quartiles of baseline age to compare dietary intake, adherence to the nutritional interventions, and CRF control. We included a total of 2278 participants with complete information on food consumption and nutrient intake, adiposity, and CRFs at baseline and after 3 years of follow-up.

### Assessments and intervention

Throughout the study, participants in the MedDiet groups had face-to-face interviews with a dietitian (yearly) and at group sessions (every 3 months) in which they received instructions and written material with information on seasonal Mediterranean foods, shopping lists, weekly meal plans, and cooking recipes for a typical week. They were instructed to follow the allocated diets, with different sessions for each intervention group. During the group sessions with dietitians and according to treatment allocation, participants in the two MedDiet groups were provided with either 1 L per week of EVOO (to consume at least 50 mL/day) or mixed nuts (30 g/day: 15 g walnuts, 7.5 g hazelnuts, and 7.5 g almonds) at no cost. Participants in the LFD group also had individual and group sessions quarterly with the dietitian, were given information and written material on the LFD (according to the American Heart Association guidelines), and received non-food gifts. None of the three groups received advice on energy restriction or promotion of physical activity.

A validated 14-point MedDiet screener was used to assess adherence to the MedDiet [20], while a nine-item questionnaire was used to evaluate adherence to the LFD [9]. Also, at baseline and yearly during follow-up, dietitians administered in face-to-face interviews a 137-item validated food-frequency questionnaire (FFQ) [21], used to assess energy and nutrients using Spanish food-composition tables [22]. The Minnesota Leisure Time Physical Activity Questionnaire and a 47-item questionnaire on education, lifestyle, history of illnesses, and medication use were also administered at baseline and yearly.

### Clinical measurements

Trained personnel performed anthropometric measurements. Height and weight of volunteers were measured using a wall‐mounted stadiometer and calibrated scales, respectively. Waist circumference was measured midway between the lowest rib and the iliac crest using an anthropometric tape. Blood pressure (BP) was measured in triplicate with a validated semiautomatic oscillometer (Omron HEM-705CP, Hoofddorp, Netherlands).

In addition, fasting blood and spot urine were obtained and plasma, serum, and buffy coats stored at − 80 °C until assay. The analytes determined in frozen samples of serum or plasma as appropriate were glucose by the glucose oxidase method, cholesterol and triglyceride by standard enzymatic procedures, high-density lipoprotein (HDL) cholesterol after precipitation with phosphotungstic acid and magnesium chloride, and low-density lipoprotein (LDL) cholesterol by the Friedewald formula when triglycerides were < 400 mg/dL.

### Ethics statement

All participants provided written informed consent to a protocol designed according to the ethical guidelines of the Declaration of Helsinki that had been approved by the institutional review boards of all participating centres. The Institutional Review Board of the Hospital Clinic (Barcelona, Spain), accredited by the US Department of Health and Human Services update for Federalwide Assurance for the Protection of Human Subjects for International (non-US) Institutions (00000738), approved the study protocol on July 16, 2002. The trial was registered (ISRCTN35739639).

### Statistical analyses

Subjects were stratified into quartiles of age. The first quartile comprised participants ≤ 62 years old (youngest) and the fourth quartile those ≥ 71 years old (oldest). Baseline characteristics of the participants are expressed as means ± SD) or percentages as appropriate. Kolmogorov and Levene tests were used to assess data normality and skewness. One-factor ANOVA with two factors (treatment group and age quartiles 1–4) was used for continuous variables and *χ*^2^ tests for categorical variables. Analysis of the effects of treatment and age quartile (Q1 vs Q4) was performed using ANOVA for analysis of the baseline visit and ANCOVA adjusted for change from baseline at 3 years. For changes in scores on the 14-item questionnaire of Mediterranean diet adherence in extreme quartiles of age, we used *χ*^2^ for comparisons between age groups, diet groups, changes between age groups, changes between diet groups. We also used the McNemar test to compare between baseline and 3-year values. Analyses were performed using SPSS 20.0. Significance level was set at *P* < 0.05.

## Results

### Study population

**Fig. 1:**
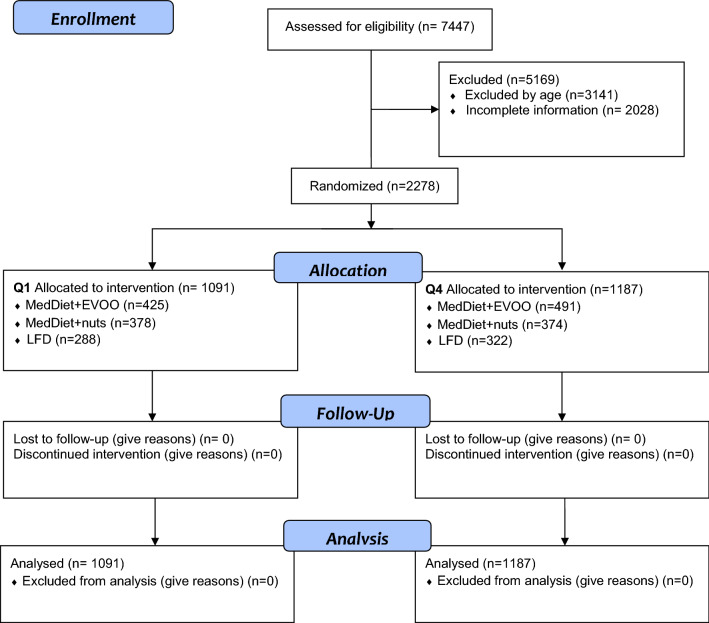
Flowchart of the study participants. The diagram includes detailed information on the participants excluded. *EVOO* extra virgin olive oil and *MedDiet* Mediterranean diet

Of the 7447 participants included, we analyzed data of 2278 with complete information on food consumption, nutrient intake, adiposity, and CRFs at baseline and after 3 years of follow-up (Fig. 1). The first (≤ 62 years, mean 59.6 ± 2.1 years) and fourth (≥ 71 years, mean 74.3 ± 2.6 years) age quartiles were composed of 1091 and 1187 subjects. Baseline characteristics of these participants are summarised in Table 1.

**Table 1:** Baseline characteristics of study subjects stratified by extreme quartiles of age

	≤ 62 years old (Q1)				≥ 71 years old (Q4)				*P* group	*P* age	*P* interaction
	All	MedDiet-EVOO	MedDiet-nuts	Control diet	All	MedDiet-EVOO	MedDiet-nuts	Control diet			
No. of subjects	1091	425	378	288	1187	491	374	322			
Age (years), mean (SD)	59.6 (2.1)	59.6 (2.2)	59.6 (2.1)	59.7 (2.1)	74.3 (2.6)	74.5 (2.6)	74.0 (2.4)	74.4 (2.5)	0.036	< 0.001	0.167
Women, *n* (%)	520 (47.7)	209 (49.2)	171 (45.2)	140 (48.6)	771 (65.0)	319 (65.0)	232 (62.0)	220 (68.3)	0.099	< 0.001	
BMI (kg/m^2^), mean (SD)	30.1 (3.9)	30.2 (3.7)	30.2 (4.1)	30.0 (4.0)	29.9 (3.8)	30.1 (3.7)	29.6 (3.8)	29.7 (3.7)	0.466	0.015	0.742
Overweight or obese (BMI ≥ 25 kg/m^2^), *n* (%)	1028 (94.2)	405 (95.3)	359 (95.0)	264 (91.7)	1083 (91.2)	455 (92.7)	334 (89.3)	294 (91.3)	0.168	0.006	
Hypertension, *n* (%)	856 (78.5)	334 (78.6)	303 (80.2)	219 (76.0)	1014 (85.4)	400 (81.5)	327 (87.4)	287 (89.1)	0.125	< 0.001	
Diabetes, *n* (%)	483 (44.3)	194 (45.6)	155 (41.0)	134 (46.5)	608 (48.8)	261 (53.2)	182 (48.7)	165 (51.2)	0.115	0.001	
Dyslipidemia, *n* (%)	816 (74.8)	311 (73.2)	290 (76.7)	215 (74.7)	812 (68.4)	326 (66.4)	262 (70.1)	224 (69.6)	0.210	0.001	
Current smoker, *n* (%)	212 (19.4)	84 (19.8)	71 (18.8)	57 (19.8)	94 (7.9)	41 (8.4)	27 (7.2)	26 (8.1)	0.925	< 0.001	
Family history of premature CHD, *n* (%)	286 (26.2)	103 (24.2)	110 (29.1)	73 (25.3)	218 (18.4)	101 (20.6)	59 (15.8)	58 (18.0)	0.899	< 0.001	
Medication, *n* (%)											
ACE inhibitors	279 (25.6)	95 (22.3)	100 (26.5)	84 (29.2)	354 (29.8)	147 (29.9)	105 (28.1)	102 (31.7)	0.204	0.024	
Statins	415 (38.0)	149 (66.2)	144 (38.1)	122 (42.4)	484 (40.8)	203 (41.3)	146 (39.0)	135 (41.9)	0.289	0.182	
Insulin	53 (4.9)	22 (5.2)	17 (4.5)	14 (4.9)	59 (5.0)	20 (4.1)	27 (7.2)	12 (3.7)	0.336	0.901	
Oral hypoglycemic drugs	282 (25.8)	109 (25.6)	91 (24.1)	82 (28.5)	369 (31.1)	159 (32.4)	112 (29.9)	98 (30.4)	0.499	0.006	
Aspirin or other antiplatelet drugs	158 (14.5)	53 (12.5)	58 (15.3)	47 (16.3)	253 (21.3)	103 (21.0)	77 (20.6)	73 (22.7) γ	0.420	< 0.001	
NSAIDS	106 (9.7)	46 (10.8)	29 (7.7)	31 (10.8)	143 (12.0)	67 (13.6)	40 (10.7)	36 (11.2)	0.120	0.075	
Antidepressants	236 (21.6)	79 (18.6)	81 (21.4)	76 (26.4)	320 (27.0)	125 (25.5)	101 (27.0)	94 (29.2)	0.044	0.003	
Diuretics	199 (18.2)	80 (18.8)	55 (14.5)	64 (22.2)	307 (25.9)	122 (24.8)	93 (24.9)	92 (28.6)	0.034	< 0.001	
Vitamins or supplements, *n* (%)	107 (9.8)	40 (9.4)	37 (9.8)	30 (10.4)	173 (14.6)	82 (16.7)	36 (9.6)	55 (17.1)	0.207	0.001	
Educational level, *n* (%)									0.087	< 0.001	
Primary school	670 (61.4)	269 (63.3)	220 (58.2)	181 (62.8)	940 (79.2)	396 (80.7)	289 (77.3)	255 (79.2)			
High school	265 (24.3)	93 (21.9)	106 (28.0)	66 (22.9)	123 (10.4)	46 (9.4)	44 (11.8)	33 (10.2)			
University	124 (9.2)	51 (12.0)	44 (11.6)	29 (10.1)	55 (4.6)	17 (4.0)	23 (6.1)	15 (4.7)			
Energy expenditure in physical activity (kcal/day), mean (SD)	256.7 (232.1)	257.4 (222.8)	262.8 (234.7)	247.4 (242.5)	228.5 (224.7)	221.3 (208.9)	245.9 (247.9)	219.1 (219.1)	0.201	0.005	0.693

Values are mean ± SD or *n* (%) as appropriate. ANOVA with two factors (group of treatment and quartiles 1–4 of age) was used for continuous variables and χ^2^-test for categorical variables

*ACE* angiotensin converting enzyme, *BMI* body mass index (calculated as weight in kilograms divided by height in square meters), *CHD* coronary heart disease, *EVOO* extra virgin olive oil, *NSAIDS* non-steroidal anti-inflammatory drug, *MedDiet* Mediterranean diet, *SD* standard deviation

In Q4, there were more women, greater prevalence of hypertension and type 2 diabetes mellitus, and lower prevalence of dyslipidemia, overweight/obesity, smoking, and family history of ischemic heart disease than participants in Q1 (*P* ≤ 0.006, all). With regard to medication, older participants took more angiotensin-converting-enzyme inhibitors, oral hypoglycaemic drugs, aspirin or other antiaggregants, antidepressants, diuretics, vitamins, or supplements than younger subjects (*P* < 0.05, all). In Q1 and Q4, participants in the control group (LFD) took more antidepressants and diuretics than those in the two MedDiet groups (*P* < 0.05, both).

### Adherence to MedDiet based on the 14-item questionnaire

As expected, both Q1 and Q4 participants allocated to the MedDiet groups significantly improved MedDiet adherence on 13 of 14 score items (Supplementary Table 1). Almost all participants (~ 97%) in the MedDiet groups used OO as their main culinary fat, whereas this percentage was lower (~ 93%) in the control group (*P* < 0.001, both). At the end of the 3-year-intervention, ≥ 75% of participants in the MedDiet groups had appropriate intake of red meat, butter, carbonated beverages, chicken, turkey, rabbit, fish, shellfish, and dishes dressed with sofrito, while optimal daily consumption of fruit and vegetables was achieved by only 60% of the participants.

These changes brought patients' diets closer to the MedDiet pattern. In fact, after 3 years of intervention, > 75% of participants allocated to the two MedDiet groups fulfilled the criteria for eight of the 14 items evaluated in our MedDiet questionnaire. Dietary improvement was lower in the control group than the MedDiet groups, and consisted mainly in decreased consumption of red meat and butter and moderate increases in vegetables, fish, and white meat (*P* < 0.05, all).

### Changes in intake of selected foods and physical activity

As shown in Table 2, at the 3-year follow-up, MedDiet-adherence scores were greater (about 1.6–2 points) in Q1 and Q4 participants in both MedDiet groups than those in the LFD group (*P* < 0.001). Adherence to the MedDiet intervention was similar between the youngest and oldest PREDIMED participants at the end of the intervention.

**Table 2:** Changes in intake of selected foods and physical activity after intervention for 3 year according to extreme quartiles of age

	≤ 62 years old (Q1)					≥ 71 years (Q4)				*P* group	*P* age	*P* interaction
	All *n* = 1091		MedDiet-EVOO *n* = 425	MedDiet-nuts *n* = 378	Control diet *n* = 288	All *n* = 1187	MedDiet-EVOO *n* = 491	MedDiet-nuts *n* = 374	Control diet *n* = 322			
Total nuts (g)												
Baseline	10.9 (10–11.7)		10.3 (8.9–11.7)^a^	12.2 (10.7–13.6)^b^	10.3 (8.6 to11.9)^ac^	10.9 (10–11.7)	9.6 (8.3–10.9)^a^	13.5 (12–14.9)^b^	9.6 (8–11.2)^ac^	< 0.001	0.972	0.315
Change 3 years	+ 5.6 (4.8–6.5)		+ 0.7 (− 0.6 to 2.1)^a^	+ 20.4 (19–21.9)^b^	− 4.3 (− 5.9 to − 2.6)^c^	+ 4.5 (3.7–5.3)	− 0.2 (− 1.5 to 1)^a^	+ 18.3 (16.9–20)^b^	− 4.6 (− 6.2 to − 3)^c^	< 0.001	0.065	0.523
EVOO (g)												
Baseline	23.1 (21.8–24.5)		23.5 (21.4–25.7)	24.3 (22–26.5)	21.6 (18.7–23.2)	19.7 (18.4–21)	19.4 (17.4–21.4)	20.9 (18.7–23.2)	18.9 (16.5–21.4)	0.166	< 0.001	0.822
Change 3 years	+ 15.7 (14.4–16.9)		+ 30.3 (28–32)^a^	+ 11.5 (9.4–14)^b^	+ 5.2 (2.8–7.6)^c^	+ 13.9 (12.7–15.1)	+ 29.2 (27–31)^a^	+ 11.5 (9.5–13.6)^b^	+ 0.9 (− 1.3 to 3.2)^c^	< 0.001	0.042	0.135
Fruits (g)												
Baseline	367 (355–379)		366 (346–385)	379 (358–399)	355 (332–379)	366 (354–378)	375 (357–393)	369 (349–390)	354 (332–376)	0.181	0.937	0.641
Change 3 years	+ 30 (20–41))		+ 44 (28–61)^a^	+ 39 (21–57)^ab^	+ 7 (− 13 to 28)^b^	+ 26 (16–36)	+ 32 (17–47)^a^	+ 26 (8–44)^ab^	+ 20 (0.3–39)^b^	0.025	0.565	0.329
Vegetables (g)												
Baseline	350 (341–358)		358 (344–372)	355 (340–369)	336 (319–353)	322 (314–330)	328 (316–341)	320 (306–335)	317 (301–333)	0.089	< 0.001	0.618
Change 3 years	+ 8.7 (0.9–16.5)		+ 9.6 (− 2.6 to 21.9)^a^	+ 23.7 (10.7–36.8)^a^	− 7.2 (− 22.4 to 7.9)^b^	− 7.3 (− 14.8 to 0.2)	+ 0.8 (− 10.5 to 12)^a^	− 0.5 (− 13.7 to 12.6)^a^	− 22.2 (− 36.4 to − 8)^b^	0.001	0.004	0.477
Legumes (g)												
Baseline	19.4 (18.7–20.2)		19.6 (18.4–20.8)	19.8 (18.5–21.2)	18.9 (17.4–20.4)	21.2 (20.4–22)	20.9 (18.8–22.1)	21.9 (20.6–23.3)	20.7 (19.3–22.2)	0.311	0.002	0.844
Change 3 years	+ 1.7 (1.1–2.3)		+ 2.7 (1.8–3.7)^a^	+ 2.5 (1.5–3.5)^a^	− 0.03 (− 1.2 to 1.1)^b^	+ 1.4 (0.9–2)	+ 2.7 (1.8–3.5)^a^	+ 2.9 (1.9–3.9)^a^	− 1.2 (− 2.3 to − 0.1)^b^	< 0.001	0.490	0.383
Fish or seafood (g)												
Baseline	106 (103.1–108.9)		106.3 (102–111)	106 (101–111)	105.6 (100–111)	95.3 (92.6–98)	95.5 (91.3–99.7)	97.3 (92.5–102.1)	93.1 (88–98.3)	0.678	< 0.001	0.765
Change 3 years	+ 4.3 (1.8–6.8)		+ 5.7 (1.8–9.6)^a^	+ 7.3 (3.1–11.5)^a^	− 0.07 (− 4.9 to 4.8)^b^	− 0.06 (− 2.5 to 2.3)	+ 2.6 (− 1 to 6.2)^a^	+ 4.4 (0.2–8.6)^a^	− 7.2 (− 11.7 to − 3)^b^	< 0.001	0.014	0.575
Meat or meat products (g)												
Baseline	145 (141.5–148)		147.1 (141.7)	149 (143.2–154.7)	138.7 (132–145)	126.5 (123–130)	124.6 (120–130)	129 (123–134.8)	125.9 (120–132)	0.095	< 0.001	0.259
Change 3 years	− 14 (− 16.6 to − 11.3)		− 14.8 (− 19 to − 10.7)	− 14 (− 18.5 to − 9.6)	− 13.1 (− 18.2 to − 8)	− 15.9 (− 18.4 to − 13)	− 11.6 (− 15.5 to − 8)	− 16 (− 20.4 to − 11.6)	− 20 (− 24.7 to − 15)	0.346	0.324	0.086
Cereals (g)												
Baseline	235.2 (229–241.6)		239.3 (229–249)	239 (228.4–249.6)	227.4 (215–240)	224.5 (218 to230.6)	224.7 (215–234)	225 (214.4–236)	223.6 (212–235)	0.427	0.016	0.577
Change 3 years	− 17.1 (− 22.5 to − 12)		− 12.6 (− 21 to − 4.2)	− 20 (− 29 to − 11)	− 18.6 (− 29 to − 8.1)	− 20 (− 25.1 to − 14.8)	− 18 (− 25.8 to − 10)	− 20.9 (− 30 to − 11.9)	− 21.1 (− 30 to − 11)	0.432	0.442	0.873
Milk and dairy products (g)												
Baseline	365.5 (353 to 380)		374.4 (353 to 396)	357.2 (335 to 380)	367.9 (342 to 394)	392.5 (380 to 405.3)	395.5 (376 to 415)	382.8 (360 to 405)	399.2 (375 to 423)	0.346	0.006	0.909
Change 3 years	− 24.8 (− 36.5 to − 13)		− 24.6 (− 43 to − 6.5)	− 14.9 (− 34.3 to 4.6)	− 35 (− 57.5 to − 12)	+ 3.3 (− 7.8 to 14.4)	+ 5.8 (− 11 to 22.7)	+ 14.3 (− 5.2 to 33.9)	− 10.2 (− 31.3 to 11)	0.101	0.001	0.960
Pastries, cakes or sweets (g)												
Baseline	23 (21.3–24.6)		23 (20.4–25.7)	22.3 (19.5–25)	23.6 (20.4–26.8)	22.2 (20.6–23.8)	21.8 (19.3–24.2)	23.4 (20.6–26.2)	21.3 (18.3–24.4)	0.947	0.510	0.489
Change 3 years	− 6.4 (− 7.7 to − 5)		− 6.6 (− 8.7 to − 4.5)	− 7.4 (− 9.6 to − 5.1)	− 5.1 (− 7.7 to − 2.5)	− 3.8 (− 5.1 to − 2.5)	− 3.7 (− 5.6 to − 1.7)	− 4.3 (− 6.6 to − 2)	− 3.4 (− 5.8 to − 0.9)	0.426	0.008	0.839
Tea (mL)												
Baseline	6.7 (5.6–7.7)		6 (4.3–7.8)	5.5 (3.7–7.4)	8.4 (6.3–10.5)	3.1 (2.1–4.2)	4.2 (2.6–5.8)	2.2 (0.3–4)	2.9 (1–4.9)	0.156	< 0.001	0.156
Change 3 years	+ 0.2 (− 0.7 to 1.1)		− 0.9 (− 2.3 to 0.6)^a^	+ 1.1 (− 0.4 to 2.7)^b^	+ 0.3 (− 1.5 to 2.1)^ab^	− 1.2 (− 2.1 to − 0.3)	− 1.8 (− 3.2 to − 0.5)^a^	0.02 (− 1.6 to 1.6)^b^	− 1.7 (− 3 to − 0.04)^ab^	0.040	0.039	0.782
Coffee (mL)												
Baseline	40.5 (37.5 to 43.4)		38.6 (33.9 to 43.3)	41 (36.1 to 46)	41.7 (36 to 47.4)	25.8 (22.9 to 28.6)	24.3 (20 to 28.7)	25.8 (20.8 to 30.8)	27.2 (21.8 to 32.5)	0.428	< 0.001	0.980
Change 3 years	+ 0.5 (− 2 to 3)		+ 0.7 (− 3.1 to 4.6)	− 2.8 (− 6.9 to 1.3)	+ 3.6 (− 1.2 to 8.3)	− 7.5 (− 9.8 to − 5.1)	− 7.4 (− 11 to − 3.8)	− 8 (− 12.1 to − 3.9)	− 7 (− 11.4 to − 2.5)	0.237	< 0.001	0.479
Alcohol (g)												
Baseline	11.7 (10.8–12.6)		11 (9.6–12.5)	12.4 (10.9–13.9)	11.7 (10–13.5)	6.6 (5.7–7.5)	7.4 (6.1–8.7)	6.9 (5.3–8.4)	5.5 (3.8–7.1)	0.464	< 0.001	0.215
Change 3 years	− 0.4 (− 1 to 0.1)		− 0.3 (− 1.1 to 0.6)	+ 0.3 (− 0.6 to 1.2)	− 1.4 (− 2.4 to − 0.3)	− 1.7 (− 2.3 to − 1.2)	− 1.5 (− 2.3 to − 0.7)	− 1.7 (− 2.6 to − 0.7)	− 2 (− 3 to − 1)	0.116	0.001	0.402
Wine (mL/day)												
Baseline	76.8 (70–83.6)		76.2 (65.4–87)	80.9 (69.4–92.3)	73.4 (60.3–86.4)	48.1 (41.6–54.7)	56.9 (46.9–66.9)	49.7 (38.2–61.2)	37.7 (25.3 to 50.1)	0.148	< 0.001	0.336
Change 3 years	− 0.2 (− 4.7 to 4.3)		+ 0.8 (− 6.2 to 7.8)	+ 6.2 (− 1.3 to 13.7)	− 7.5 (− 16.2 to 1.1)	− 8.1 (− 12.4 to − 3.9)	− 4.6 (− 11 to 1.9)	− 8.8 (− 16.3 to − 1.3)	− 11.1 (− 19.3 to − 3)	0.090	0.012	0.290
14− item score												
Baseline	8.7 (8.5–8.7)		8.8 (8.6–8.9)^a^	8.8 (8.6–9)^a^	8.4 (8.2–8.6)^b^	8.7 (8.5–8.8)	8.7 (8.5–8.9)^a^	8.7 (8.6–8.9)^a^	8.5 (8.3–8.7)^ab^	0.006	0.988	0.786
Change 3 years	+ 1.4 (1.3–1.5)		+ 1.8 (1.6–2)^a^	+ 2.1 (1.9–2.3)^b^	+ 0.3 (0.1–0.5)^c^	+ 1.3 (1.2–1.4)	+ 1.6 (1.4–1.7)^a^	+ 2 (1.9–2.2)^b^	+ 0.2 (0.02–0.4)^c^	< 0.001	0.137	0.579
Physical activity (kcal/day)												
Baseline	255.9 (242–270)		257 (236–279)	262.8 (239.8–286)	247.4 (221–274)	228.7 (216–242)	221.3 (201–241)	245.9 (223–269)	219.1 (194–244)	0.201	0.005	0.693
Change 3 years	+ 47.9 (33.4–62)		+ 43.2 (20.3–66.1)	+ 43.1 (18.8–67.4)	+ 57.3 (29.5–85)	− 0.8 (− 14.8 to 13.1)	+ 17.8 (− 3.5 to 39)	+ 5.7 (− 18.7 to 30.2)	− 25.1 (− 52 to 0.2)	0.498	< 0.001	0.062

Values are expressed as mean (95% CI). The analysis of the effect of the treatment group and the Quartile of Age (Q1 vs Q4) were performed by ANOVA for the analysis of the baseline visit and by ANCOVA adjusted with the baseline values for the change at 3 years

*EVOO* extra virgin olive oil, *MedDiet + EVOO* Mediterranean diet supplemented with extra virgin olive oil, *MedDiet + Nuts* Mediterranean diet supplemented with nuts

^a,b,c^Treatment groups with different superscript letters show statistical differences according to the Bonferroni correction

In addition, the two MedDiet groups showed good adherence to the supplemented foods (EVOO or nuts, *P* ≤ 0.001 for both), which was slightly higher in younger than older (*P* ≤ 0.065, both) participants. Both the Q1 and Q4 groups showed increased consumption of fruit and legumes and decreased consumption of meat and meat products and cereals. Consumption of pastries, cakes, sweets, and alcohol had significantly reduced in both groups (Q1 and Q4, *P* ≤ 0.012, all). In comparison with Q4, participants in Q1 showed significantly increased consumption of vegetables, fish, seafood, tea, and coffee and significantly decreased consumption of milk and dairy products (*P* < 0.05, all).

However, the MedDiet groups in Q1 and Q4 differed in some specific foods. As such, these groups reported higher consumption of fruit, legumes, fish and seafood than the control group (*P* ≤ 0.025, all). In addition, the Q1 group disclosed higher consumption of vegetables and tea (*P* < 0.05, both) than the Q4 group, while the latter group had higher consumption of milk and dairy products (*P* = 0.032).

Finally, only participants in the Q1 group had significantly increased their physical activity during follow-up, which was significantly higher than those in Q4 (*P* < 0.001). On the other hand, Q4 participants in the control group had significantly decreased physical activity (*P* < 0.05).

### Changes in energy and nutrient intake in young and old cohorts

The Q1 and Q4 groups had significantly decreased consumption of cholesterol and calcium at the end of the study (*P* ≤ 0.007). Participants in Q1 had increased intake of magnesium in comparison to Q4 (*P* = 0.051), while participants in Q4 had higher reductions in sodium than participants in Q1 (*P* = 0.08). On the other hand, consumption of polyunsaturated fatty acids (PUFAs; *P* < 0.001), α-linolenic acid (*P* < 0.001), and marine *n*-3 FAs (*P* ≤ 0.002) had increased during the study period in both quartiles (Table 3).

**Table 3:** Changes in intake of energy and nutrients after 3 years stratified by extreme quartiles of age

	≤ 62 years old (Q1)				≥ 71 years (Q4)				*P* group	*P* age	*P* interaction
	All *n* = 1091	MedDiet-EVOO *n* = 425	MedDiet-nuts *n* = 378	Control diet *n* = 288	All *n* = 1187	MedDiet-EVOO *n* = 491	MedDiet-nuts *n* = 374	Control diet *n* = 322			
Total energy, Kcal/day											
Baseline	2343(2307–2377)	2379(2324–2434)^a^	2376(2318–2435)^a^	2272(2205–2339)^b^	2182(2149–2216)	2193(2141 to2244)^a^	2221(2162–2279)^a^	2133(2070–2196)^b^	0.005	< 0.001	0.717
Change 3 years	− 85.6 (− 114 to − 58)	− 29.1 (− 73 to 15)	+ 6.7 (− 40 to 54)	− 235 (− 290 to − 181)	− 110 (− 137 to − 83)	− 47.9 (− 89 to − 7)	− 0.04 (− 47 to 47)	− 282 (− 333 to − 231)	< 0.001	0.228	0.727
Total protein (g)											
Baseline	96.3 (95–97.6)	97.6 (95.6–99.7)^ab^	97.7 (95.5–99.9)^a^	93.6 (91.1–96.1)^b^	89.8 (88.6–91.1)	89.8 (87.9–91.7)^ab^	90.8 (88.6–93)^a^	88.9 (86.5–91.3)^b^	0.028	< 0.001	0.392
Change 3 years	− 3.59 (− 4.7 to − 2.5)	− 3.6 (− 5.3 to − 1.9)^a^	+ 0.17 (− 1.7 to 2)^b^	− 7.28 (− 9.4 to − 5.2)^c^	− 4.66 (− 5.7 to − 3.6)	− 3.4 (− 5 to − 1.8)^a^	− 0.8 (− 2.7 to 1)^ab^	− 9.8 (− 11.7 to − 7.8)^c^	< 0.001	0.162	0.346
Total carbohydrate (g)											
Baseline	242 (237–247)	247 (239–254)	245 (237–253)	234 (225–243)	231 (227–236)	233 (226–240)	232 (224–240)	228 (219–237)	0.077	0.001	0.593
Change 3 years	− 15.7 (− 19.5 to − 11.8)	− 12.5 (− 18 to − 6.5)	− 13.8 (− 20.2 to − 7.4)	− 20.7 (− 28 to − 13)	− 14.7 (− 18.4 to − 11.1)	− 13 (− 18.5 to − 7.5)	− 11.3 (− 17.7 to − 5)	− 19.9 (− 26.8 to − 13)	0.039	0.730	0.890
Fibre (g)											
Baseline	25.5 (25–26)	25.8 (25–26.6)	26.1 (25.2–27)	24.7 (23.7–25.7)	24.7 (24.2–25.1)	24.9 (24.2–25.7)	24.9 (24–25.8)	24.1 (23.2–25.1)	0.039	0.016	0.809
Change 3 years	+ 0.58 (0.14–1.02)	+ 0.89 (0.2–1.6)^a^	+ 1.8 (1.1–2.5)^b^	− 0.95 (− 1.8 to − 0.1)^c^	+ 0.12 (− 0.3 to 0.5)	+ 0.36 (− 0.3 to 1)^a^	+ 1.46 (0.72–2.2)^b^	− 1.47 (− 2.27 to − 0.67)^c^	< 0.001	0.133	0.960
Total fat (g)											
Baseline	100.8 (99.1–102.6)	102.7 (100–105.5)^a^	102 (99.1–104.9)^a^	97.8 (94.5–101.2)^b^	94.7 (93–96.4)	94.4 (91.9–97)^a^	97.8 (94.9–100.7)^a^	91.9 (88.7–95)^b^	0.005	< 0.001	0.346
Change 3 years	− 0.99 (− 2.4 to 0.4)	+ 4.04 (1.8–6.3)^a^	+ 6.21 (3.8–8.6)^b^	− 13.2 (− 16 to − 10.5)^c^	− 1.97 (− 3.3 to − 0.6)	+ 3.25 (1.2–5.3)^a^	+ 7.1 (4.7–9.5)^b^	− 16.25 (− 19 to − 13.6)^c^	< 0.001	0.337	0.320
SFA (g)											
Baseline	26.2 (25.6–26.7)	26.6 (25.7–27.4)	26.4 (25.5–27.3)	25.6 (24.5–26.6)	24.4 (23.9–24.9)	24.5 (23.7–25.3)	25 (24.1–25.9)	23.7 (22.7–24.7)	0.068	< 0.001	0.717
Change 3 years	− 2.99 (− 3.4 to − 2.6)	− 2.46 (− 3.1 to − 1.8)^a^	− 1.92 (− 2.6 yo − 1.2)^a^	− 4.61 (− 5.4 to − 3.8)^b^	− 2.56 (− 3 to − 2.17)	− 1.46 (− 2 to − 0.9)^a^	− 1.27 (− 2 to − 0.6)^a^	− 4.97 (− 5.7 to − 4.2)^b^	< 0.001	0.136	0.151
MUFA (g)											
Baseline	49.8 (48.9–50.7)	51.2 (49.7–52.6)^a^	50.1 (48.6–51.7)^a^	48.1 (46.3–49.9)^b^	46.9 (46–47.8)	47.1 (45.8–48.5)^a^	48.2 (46.7–49.7)^a^	45.3 (43.7–47)^b^	0.003	< 0.001	0.344
Change 3 years	+ 1.53 (0.7–2.3)	+ 5.96 (4.7–7.2)^a^	+ 3.92 (2.6–5.2)^a^	− 5.31 (− 6.8 to − 3.8)^ab^	+ 1.01 (0.3–1.8)	+ 4.95 (3.8–6.1)^a^	+ 4.76 (3.4–6.1)^a^	− 6.68 (− 8.1 to − 5.3)^b^	< 0.001	0.351	0.220
PUFA (g)											
Baseline	16.1 (15.6–16.5)	16 (15.3–16.6)^a^	16.7 (16–17.3)^b^	15.5 (14.7–16.3)^a^	15.2 (14.8–15.6)	14.7 (14.1–15.3)^a^	16.2 (15.5–16.9)^b^	14.7 (14–15.4)^a^	< 0.001	0.003	0.496
Change 3 years	+ 0.42 (0.07–0.76)	+ 0.01 (− 0.5 to 0.6)^a^	+ 3.82 (3.2–4.4)^b^	− 2.58 (− 3.25 to − 1.9)^c^	− 0.06 (− 0.39 to 0.28)	− 0.3 (− 0.8 to 0.2)^a^	+ 3.5 (2.9–4.1)^b^	− 3.38 (− 4 to − 2.7)^c^	< 0.001	< 0.001	0.668
Linoleic acid (g/day)											
Baseline	13.3 (12.9–13.7)	13.2 (12.6–13.8)^a^	13.8 (13.2–14.4)^b^	12.8 (12.1–13.6)^a^	12.6 (12.3–13)	12.2 (11.6–12.7)^a^	13.5 (12.9–14.2)^b^	12.1 (11.5–12.8)^a^	0.001	0.012	0.445
Change 3 years	+ 0.28 (− 0.03 to 0.6)	− 0.15 (− 0.6 to 0.3)^a^	3.27 (2.8–3.8)^b^	− 2.28 (− 2.9 to − 1.7)^c^	0.01 (− 0.28 to 0.3)	− 0.28 (− 0.7 to 0.2)^a^	3.13 (2.6–3.66)^b^	− 2.81 (− 3.4 to − 2.2)^c^	< 0.001	0.223	0.724
α-Linolenic acid (g/day)											
Baseline	1.43 (1.39–1.48)	1.45 (1.38–1.52)^a^	1.49 (1.42–1.57)^b^	1.36 (1.28–1.45)^a^	1.38 (1.33–1.42)	1.33 (1.26–1.39)^a^	1.51 (1.43–1.58)^b^	1.29 (1.21–1.38)^a^	< 0.001	0.053	0.173
Change 3 years	+ 0.097(0.05–0.1)	+ 0.01(− 0.06 to 0.08)^a^	+ 0.55 (0.48–0.62)^b^	− 0.27(− 0.35 to − 0.18)^c^	+ 0.04(− 0.003 to 0.08)	+ 0.01(− 0.06 to 0.06)^a^	+ 0.48 (0.41–0.55)^b^	− 0.37 (− 0.44 to − 0.29)^c^	< 0.001	< 0.001	0.422
Marine n-3 fatty acids (g/day)											
Baseline	0.84 (0.82–0.87)	0.86 (0.82–0.91)	0.82 (0.78–0.87)	0.85 (0.79–0.9)	0.76 (0.74–0.79)	0.77 (0.73–0.81)	0.78 (0.73–0.82)	0.74 (0.69–0.79)	0.542	< 0.001	0.500
Change 3 years	+ 0.06 (0.03–0.09)	+ 0.1 (0.06–0.14)^a^	+ 0.12 (0.07–0.16)^a^	− 0.04 (− 0.09 to 0.01)^b^	+ 0.002 (− 0.02 to 0.03)	+ 0.05 (0.01–0.09)^a^	+ 0.05 (0.01–0.1)^a^	− 0.09 (− 0.14 to − 0.05)^b^	< 0.001	0.002	0.954
Cholesterol (g)											
Baseline	384.1 (377–391)	383.5 (372–395)^ab^	395.8 (384–408)^a^	373.1 (359–387)^b^	349.2 (342–356)	347.9 (337–359)^ab^	357.8 (346–370)^a^	341.9 (329–355)^b^	0.012	< 0.001	0.872
Change 3 years	− 28.1 (− 33.9 to − 22.2)	− 23.7(− 32.7 to − 14.6)^a^	− 23.7(− 33.4 to − 13.9)^a^	− 36.9(− 48.1 to − 25.7)^b^	− 26.1 (− 31.7 to − 20.5)	− 17.7 (− 26.2 to − 9.3)^a^	− 18.6 (− 28.3 to − 8.9)^a^	− 41.9 (− 52.4 to − 31.4)^b^	0.628	< 0.001	0.511
Magnesium (mg)											
Baseline	386.1 (380–392)	389 (379–399)^ab^	393.1 (383–403)^a^	376.1 (364–388)^b^	364 (358–369)	365.1 (356–374)^ab^	368.4 (358–379)^a^	357 (346–368)^b^	0.032	< 0.001	0.860
Change 3 years	+ 0.34 (− 4.7 to 5.4)	− 2.75 (− 10.6 to 5.1)^a^	+ 28.7 (20.2–37.1)^b^	− 24.9(− 34.6 to − 15.2)^c^	− 6.64 (− 11.5 to − 1.81)	− 6.11 (− 13.4 to 1.2)^a^	+ 22.9 (14.4–31.3)^b^	− 36.7 (− 45.8 to − 27.5)^c^	< 0.001	0.051	0.624
Potassium (mg)											
Baseline	4424 (4358–4490)	4474 (4370–4578)^a^	4512 (4402–4622)^a^	4286 (4160–4412)^b^	4240 (4177–4303)	4272 (4175–4369)^a^	4298 (4187–4408)^a^	4150 (4031–4270)^b^	0.004	< 0.001	0.778
Change 3 years	− 35.7 (− 91.3 to 19.9)	− 18.1 (− 105 to 68.8)^a^	+ 151 (57.9–244)^b^	− 240 (− 348 to − 132)^c^	− 97 (− 150 to − 43.8)	− 30.2 (− 111 to 51)^a^	+ 67.4 (− 26 to 161)^b^	− 328 (− 429 to − 227)^c^	< 0.001	0.119	0.644
Sodium (mg)											
Baseline	2541 (2488–2595)	2594 (2509–2679)	2566 (2476–2656)	2464 (2361–2567)	2274 (2222–2325)	2266 (2187–2345)	2313 (2223–2404)	2242 (2145–2340)	0.153	< 0.001	0.484
Change 3 years	− 253 (− 294 to − 212)	− 248 (− 311 to − 184)^a^	− 218 (− 286 to − 151)^a^	− 293 (− 371 to − 215)^b^	− 303 (− 342 to − 254)	− 262 (− 320 to − 203)^a^	− 263 (− 331 to − 195)^a^	− 385 (− 458 to − 311)^b^	0.016	0.080	0.542
Calcium (mg)											
Baseline	1045 (1023–1068)	1062 (1027–1098)	1048 (1010–1085)	1026 (983–1069)	1033 (1011–1054)	1046 (1013–1079)	1025 (987–1063)	1027 (987–1068)	0.330	0.434	0.827
Change 3 years	− 56.1 (− 74.6 to − 37.5)	− 58.5(− 87.4 to − 29.4)^a^	− 14.6 (− 45.7 to 16.4)^a^	− 95.1 (− 131 to − 59.1)^b^	− 28.8 (− 46.5 to − 11)	− 18.8 (− 45.8 to 8.1)^a^	+ 5.4 (− 25.8 to 36.6)^a^	− 73 (− 106.7 to − 39.2)^b^	0.002	0.007	0.775

Values are expresed as mean (95% CI). The analysis of the effect of the treatment group and the Quartile of Age (Q1 vs Q4) were performed by ANOVA for the analysis of the baseline visit and by ANCOVA adjusted with the baseline values for the change at 3 years

*EVOO* extra virgin olive oil, *MedDiet + EVOO* Mediterranean diet supplemented with extra virgin olive oil, *MedDiet + Nuts* Mediterranean diet supplemented with nuts, *MUFA* monounsaturated fat acids, *PUFA* polyunsaturated fat acids and *SFA* saturated fat acid

^a,b,c^Treatment groups with different superscript letters show statistical differences according to the Bonferroni correction

Q1 and Q4 subjects in the two MedDiet groups had reduced consumption of protein intake, total carbohydrates, saturated FAs (SFAs), potassium, and sodium (*P* < 0.05, all) and increased consumption of fibre, monounsaturated FAs (MUFAs), α-linolenic acid, and marine *n*-3 FAs (*P* < 0.001, all). In addition, Q1 and Q4 participants on the MedDiet supplemented with nuts had increased consumption of α-linolenic acid and magnesium (*P* < 0.001, both). On the other hand, for the LFD in both Q1 and Q4, there were significant reductions in energy, total fat, and calcium intake (*P* < 0.001). Finally, Q1 participants in the two MedDiet groups showed significantly increased PUFAs (*P* < 0.001).

### Changes in classical cardiovascular risk factors

**Fig. 2:**
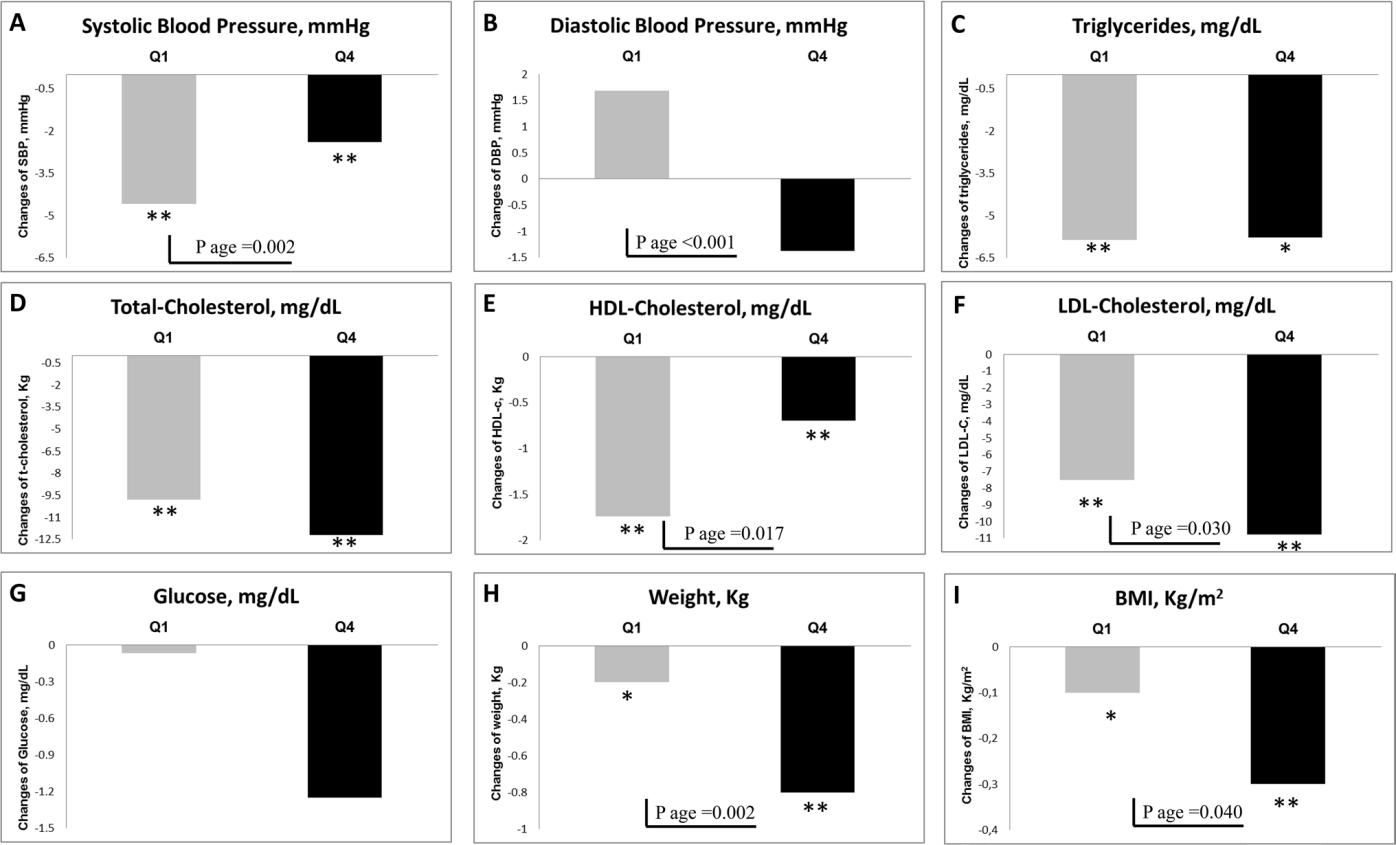
Changes in cardiovascular risk factors after 3 years of nutritional nutrition stratified by extreme quartiles of age. The analysis of the effect of the treatment group and the Quartile of Age (Q1 vs Q4) were performed by ANOVA for the analysis of the baseline visit and by ANCOVA adjusted with the baseline values for the change at 3 years. *P < 0.05 and **P < 0.01 indicates statistical significance by *t*-test for related samples. *BMI* body mass index, *DBP* diastolic blood pressure, *HDL* high density lipoprotein, *LDL* low density lipoprotein, *SBP* systolic blood pressure

As represented in Fig. 2 and Supplementary Table 2, systolic BP and HDL-cholesterol levels had decreased in both quartiles (*P* ≤ 0.002, both) at 3 years, although the change was greater in the Q1 group. In addition, modest reductions in body weight and (*P* < 0.05) occurred in both quartiles, slightly superior in Q4 (*P* ≤ 0.04). Likewise, a reduction in serum LDL-cholesterol (*P* = 0.030) was observed in Q1 and Q4 subjects, although this reduction was higher in the Q4 group. Finally, it is noteworthy that diastolic BP had decreased in Q4, contrary to Q1, whose BP had increased (*P* < 0.001 and *P*_interaction_ = 0.01). No significant differences were observed in serum triglycerides, fasting blood glucose, or waist circumference.

## Discussion

The present results show that adherence to the nutritional intervention implemented in the PREDIMED trial was similar between older (Q4, ≥ 71 years old) and younger (Q1, ≤ 62 years old) participants and was maintained throughout a 3-year follow-up. Both cohorts had increased their scores on the 14-item MedDiet questionnaire by 1.6–2 points at the end of the intervention. As a result, improvement in CRF control was also similar in the two groups. In concordance, subgroup analyses on the primary outcome of the PREDIMED trial also revealed similar CVD-risk reduction with the MedDiet in participants aged < 70 years and ≥ 70 years [9].

While comparisons of dietary intake and MedDiet adherence with younger participants was not possible due to inclusion criteria (aged 55–80 years), some differences has been observed. Although younger and older subjects reached similar overall adherence to the MedDiet (10.1 vs 10.0 of 14 points, respectively), some between-group differences deserve to be mentioned. At the end of the intervention, a lower proportion of Q4 participants had achieved recommended doses of OO, nuts, fruit, legumes, and commercial sweets, while a higher percentage had reached recommended doses of red meat, dietary milk, and alcohol than Q1 participants.

The National Health and Nutrition Examination Survey and other epidemiological studies have revealed that intake of beneficial nutrients, such as complex carbohydrates, fibre, MUFAs, and PUFAs, is reduced in older individuals, while intake of unhealthy nutrients, such as SFAs, is increased [23–25]. Data from our study showed that independently of age, allocation to the two MedDiet groups resulted in significant reductions in SFA intake and increased intake of MUFA and fibre, while PUFA intake increased only in participants allocated to the MedDiet supplemented with nuts. Higher fibre intake can be related to healthy dietary changes, such as higher consumption of fruit, vegetables, legumes, and nuts. In fact, higher intake of MUFAs, PUFAs, and fibre can have a protective effect against such age-related disorders as cognitive decline, CVD, diabetes, and cancer, as well as development of frailty [8–11, 18, 26–30]. Furthermore, it should be noted that while both MedDiet groups from the Q4 group significantly increased marine ω_3_ FA intake, only those allocated to the MedDiet supplemented with nuts significantly increased α-linolenic intake, as expected from the richness of walnuts in this FA. Importantly, intake of long-chain PUFAs, such as marine *ω*_3_ FAs and α-linolenic acid, is associated with improved cognition and reduced risk of Alzheimer’s disease and other dementias [28, 29, 31].

Ageing is frequently associated with malnutrition, particularly in frail individuals, due to such factors as hyporexia, decreased saliva production, disturbances in taste and smell, and biological changes, such as alterations in ghrelin and cholecystokinin production, as well as polypharmacy effects [14, 16]. Epidemiological studies have shown that compared to young people, the average daily caloric intake is lower in the elderly by approximately 1000 and 700 kcal/day in men and women, respectively [24]. Also, an observational study reported that a significant proportion of persons aged > 80 years had a daily energy intake < 20 kcal/kg body weight [32]. However, in our study, daily energy intake at the end of the study was lower in Q1 and Q4 participants in the LFD group than the two MedDiet groups, whose intake of protein and fat was maintained or even increased. In other reports on elderly people, animal-protein intake was reduced, which was attributed in part to difficulty chewing and swallowing [33]. It is well established that old people must maintain adequate daily protein intake as a preventive measure to preserve skeletal muscle mass and avoid sarcopenia and frailty [34].

As stated in most guidelines, non-pharmacological measures are the first therapeutic approach to improve control of CRFs and reduce incidence of CVD [5, 12, 13]. However, there is concern whether elderly persons are capable of improving unhealthy lifestyle habits and maintaining beneficial changes in the long run, probably because changes associated with ageing, such as loss of appetite (reduced taste and smell), loneliness, eating alone, depression, and low income, can influence food choices and dietary habits [14–16]. The usefulness of non-pharmacological measures to reduce CVD incidence or improve nutritional status in elderly people has recently been reviewed [35]. While the evidence is of low quality, because it was based on heterogeneous results obtained from small cohorts with short follow-up periods [35–42], the results are encouraging. Adherence to a MedDiet intervention for 6 months in a cohort of 166 elders (mean age 71 years) was high (85%) and associated with lower BP and improvement in endothelial function [36]. Likewise, adherence to a DASH diet for ≤ 3 months in two cohorts of aged Asian individuals was also high and resulted in lower BP [37–39]. Also, a multicomponent nutritional telemonitoring intervention applied for 6 months in elderly people (mean age 78 years) at risk of undernutrition showed good adherence and resulted in improved diet quality and nutritional status [40]. The NU-AGE project conducted on a cohort of 1141 elderly European subjects demonstrated that it is possible to change dietary habits of elders towards a healthier diet that can improve cognitive and bone health [41]. On the other hand, regarding physical activity, preliminary data from the PREDIMED-Plus study have revealed that it is also feasible to increase physical activity in old people in the long run (12 months) [42], which confirms findings from a recent meta-analysis [43]. Other studies, however, have shown negative results [44, 45].

Our study has strengths, such as the clinical trial design, repeated data collection, validated FFQ, standardized measurements of clinical and nutritional variables, a relatively large sample (2200 patients), and long-follow-up (3 years). The main limitations are the use of the FFQ may have led to a misclassification of the exposure due to an overestimation of food intake and the fact that dietary data are self-reported. In addition, self-reported questionnaires about diet, physical activity and other medical data can lead to misclassification, which would attenuate the association of the exposure variables with the outcome. Furthermore, potential residual confounding and the lack of generalizability of the results to other populations are limitations in this study. Unmeasured confounders may have distorted results for predictors of dietary adherence, though analyses were adjusted for a wide array of confounders, and a strong confounder unrelated to these characteristics is unlikely. Finally, the findings in our Mediterranean cohort of individuals at high cardiovascular risk cannot easily be extrapolated to other populations.

## Conclusion

We report that persons aged > 70 years can improve their dietary habits and adhere in the long term to an enhanced MedDiet in a similar way to younger adult individuals. This goal was reached in part because participants were taught and trained with high intensity by motivated dietitians and received key MedDiet foods for free. As a healthy and high-quality diet, the MedDiet was associated with reduced potency of CRFs to a similar extent in elderly and younger individuals. The benefits of the MedDiet for non-communicable diseases include reduced rates of diabetes and some cancers, lower BP, and improved cognition, as described in other PREDIMED reports [18, 19, 29, 30]. The take-home message is that we should not miss the opportunity to apply such non-pharmacological measures as the MedDiet, which has high efficacy without adverse effects, to improve the overall health of aged people. It is never too late to change dietary habits to achieve healthy ageing.

## Supplementary Information

Below is the link to the electronic supplementary material.

Supplementary file1 (DOCX 36 KB)

## Abbreviations

BMI: Body-mass index
CHD: Coronary heart disease
CRF: Cardiovascular risk factor
CVD: Cardiovascular disease
EVOO: Extra-virgin olive oil
FFQ: Food-frequency questionnaire
HDL: High-density lipoprotein
LDL: Low-density lipoprotein
LFD: Low-fat diet
MedDiet: Mediterranean diet
MUFAs: Monounsaturated fatty acids
PUFAs: Polyunsaturated fatty acids
PREDIMED: Prevention with Mediterranean Diet
SFAs: Saturated FAs

## Declarations

### Availability of data and materials

The dataset analyzed during the current study is not publicly available due to national data regulations and for ethical reasons, e.g. we do not have the explicit written consent of the study volunteers to make their deidentified data available at the end of the study. However, data described in the manuscript, codebook, and analytic codes will be made available upon request by contacting the PREDIMED Steering Committee (predimed-steering-committe@googlegroups.com). The request will be passed to all the members of the committee for deliberation.
