# Supplementary material for: Change to a healthy diet in people over 70 years old: the PREDIMED experience
Source: Eur J Nutr. 2021 Nov 28;61(3):1429–44. doi: 10.1007/s00394-021-02741-7 (PMC8921045; doi:10.1007/s00394-021-02741-7)
Supplement: Supplementary file 1 — Supplementary file1 (DOCX 36 KB) [file 394_2021_2741_MOESM1_ESM.docx]

**Supplementary Table 1. Scoring of the 14-item questionnaire of Mediterranean diet adherence in extreme quartiles of age**

|  |  | All | | | **≤62 years old (Q1)** | | | | **≥71 years old (Q4)** | | | |
| --- | --- | --- | --- | --- | --- | --- | --- | --- | --- | --- | --- | --- |
|  |  | **Q1** N= 1091 | **Q4** N= 1187 | *P*^1^ Age | MedDiet-EVOO  N= 378 | MedDiet-nuts  N= 288 | Control diet  N= 425 | *P*^2^ group | MedDiet-EVOO  N= 491 | MedDiet-nuts  N= 374 | Control diet  N= 322 | *P*^2^ group |
|  | Criteria for  1 point | n with  1 point (%) | n with  1 point (%) |  | n with  1 point (%) | n with  1 point (%) | n with  1 point (%) |  | n with  1 point (%) | n with  1 point (%) | n with  1 point (%) |  |
| 1. Do you use olive oil as main culinary fat? | Yes |  |  |  |  |  |  |  |  |  |  |  |
| Baseline |  | 995 (91.2) | 1081 (91.1) | 0.913 | 397 (93.4) | 341 (90.2) | 257 (89.2) | 0.109 | 450 (91.6) | 341 (91.2) | 290 (90.1) | 0.737 |
| 3 years |  | 1059 (97.1) | 1140 (96.0) | 0.181 | 421 (99.1) | 370 (97.9) | 268 (93.1) | <0.001 | 484 (98.6) | 361 (96.5) | 295 (91.6) | <0.001 |
| Change | *P*^3^ | <0.001 | <0.001 | 0.524^4^ |  |  |  | 0.001^5^ |  |  |  | <0.001^5^ |
| 2. How much olive oil do you consume in given day (including oil used for frying, salads, out-of-house meals, etc.)? | ≥4 tbsp |  |  |  |  |  |  |  |  |  |  |  |
| Baseline |  | 789 (72.3) | 831 (70.0) | 0.224 | 323 (76.0) | 267 (70.6) | 199 (69.1) | 0.086 | 350 (71.3) | 263 (70.3) | 218 (67.7) | 0.545 |
| 3 years |  | 842 (77.2) | 892 (75.1) | 0.256 | 403 (94.8) | 283 (74.9) | 156 (54.2) | <0.001 | 451 (91.8) | 288 (77.0) | 153 (47.5) | <0.001 |
| Change | *P*^3^ | 0.007 | 0.003 | 0.993^4^ |  |  |  | <0.001^5^ |  |  |  | <0.001^5^ |
| 3. How many vegetable servings do you consume per day? (1serving: 200g [consider side dishes as half serving]) | ≥2* |  |  |  |  |  |  |  |  |  |  |  |
| Baseline |  | 501 (45.9) | 464 (39.1) | 0.001 | 196 (46.1) | 189 (50.0) | 116 (40.3) | 0.044 | 206 (42.0) | 147 (39.3) | 111 (34.5) | 0.101 |
| 3 years |  | 724 (66.4) | 733 (61.8) | 0.022 | 301 (70.8) | 250 (66.1) | 173 (60.1) | 0.012 | 311 (63.3) | 248 (66.3) | 174 (54.0) | 0.003 |
| Change | *P*^3^ | <0.001 | <0.001 | 0.211^4^ |  |  |  | 0.117^5^ |  |  |  | 0.015^5^ |
| 4. How many fruit units (including natural fruit juices) do you consume per day? | ≥3 |  |  |  |  |  |  |  |  |  |  |  |
| Baseline |  | 546 (50.0) | 614 (51.7) | 0.423 | 215 (50.6) | 204 (54.0) | 127 (44.1) | 0.040 | 246 (50.1) | 207 (55.3) | 161 (50.0) | 0.238 |
| 3 years |  | 661 (60.6) | 709 (59.7) | 0.667 | 264 (62.1) | 244 (64.6) | 153 (53.1) | 0.008 | 285 (58.0) | 245 (65.5) | 179 (55.6) | 0.018 |
| Change | *P*^3^ | <0.001 | <0.001 | 0.499^4^ |  |  |  | 0.989^5^ |  |  |  | 0.223^5^ |
| 5. How many servings of red meat, hamburger, or meat products (ham, sausage, etc.) do you consume per day? (1 serving: 100–150 g) | <1 |  |  |  |  |  |  |  |  |  |  |  |
| Baseline |  | 893 (81.9) | 1056 (89.0) | <0.001 | 355 (83.5) | 311 (82.3) | 227 (78.8) | 0.268 | 438 (89.2) | 334 (89.3) | 284 (88.2) | 0.876 |
| 3 years |  | 1023 (93.8) | 1141 (96.1) | 0.010 | 398 (93.6) | 356 (94.2) | 269 (93.4) | 0.911 | 473 (96.3) | 361 (96.5) | 307 (95.3) | 0.688 |
| Change | *P*^3^ | <0.001 | <0.001 | <0.001^4^ |  |  |  | 0.762^5^ |  |  |  | >0.999^5^ |
| 6. How many servings of butter, margarine, or cream do you consume per day? (1 serving: 12 g) | <1 |  |  |  |  |  |  |  |  |  |  |  |
| Baseline |  | 1002 (91.8) | 1072 (90.3) | 0.201 | 396 (93.2) | 349 (92.3) | 257 (89.2) | 0.154 | 447 (91.0) | 332 (88.8) | 293 (91.0) | 0.476 |
| 3 years |  | 1055 (96.7) | 1137 (95.8) | 0.254 | 415 (97.6) | 361 (95.5) | 279 (96.9) | 0.232 | 482 (98.2) | 356 (95.2) | 299 (92.9) | 0.001 |
| Change | *P*^3^ | <0.001 | <0.001 | 0.488^4^ |  |  |  | 0.384^5^ |  |  |  | 0.025^5^ |
| 7. How many sweet or carbonated beverages do you drink per day? | <1 |  |  |  |  |  |  |  |  |  |  |  |
| Baseline |  | 958 (87.8) | 1076 (90.6) | 0.029 | 376 (88.5) | 330 (87.3) | 252 (87.5) | 0.865 | 446 (90.8) | 338 (90.4) | 292 (90.7) | 0.973 |
| 3 years |  | 1003 (91.9) | 1107 (93.3) | 0.226 | 388 (91.3) | 352 (93.1) | 263 (91.3) | 0.577 | 459 (93.5) | 352 (94.1) | 296 (91.9) | 0.499 |
| Change | *P*^3^ | 0.001 | 0.009 | 0.020^4^ |  |  |  | 0.772^5^ |  |  |  | 0.848^5^ |
| 8. How much wine do you drink per week? | ≥7 glasses |  |  |  |  |  |  |  |  |  |  |  |
| Baseline |  | 382 (35.0) | 322 (27.1) | <0.001 | 148 (34.8) | 137 (36.2) | 97 (33.7) | 0.785 | 139 (28.3) | 105 (28.1) | 78 (24.2) | 0.389 |
| 3 years |  | 364 (33.4) | 328 (27.6) | 0.003 | 142 (33.4) | 137 (36.2) | 85 (29.5) | 0.189 | 147 (29.9) | 109 (29.1) | 72 (22.4) | 0.045 |
| Change | *P*^3^ | 0.229 | 0.718 | 0.192^4^ |  |  |  | 0.193^5^ |  |  |  | 0.353^5^ |
| 9. How many servings of legumes do you consume per week? (1 serving : 150g) | ≥3 |  |  |  |  |  |  |  |  |  |  |  |
| Baseline |  | 242 (22.2) | 292 (24.6) | 0.173 | 93 (21.9) | 83 (22.0) | 66 (22.9) | 0.940 | 117 (23.8) | 92 (24.6) | 83 (25.8) | 0.820 |
| 3 years |  | 396 (36.3) | 435 (36.6) | 0.862 | 165 (38.8) | 152 (40.2) | 79 (27.4) | 0.001 | 194 (39.5) | 155 (41.4) | 86 (26.7) | <0.001 |
| Change |  | <0.001 | <0.001 | 0.386^4^ |  |  |  | 0.006^5^ |  |  |  | 0.001^5^ |
| 10. How many servings of fish or shellfish do you consume per week? (1 serving 100–150g of fish or 4–5 units or 200g of shellfish) | ≥3 |  |  |  |  |  |  |  |  |  |  |  |
| Baseline |  | 622 (57.0) | 666 (56.1) | 0.664 | 245 (57.6) | 214 (56.6) | 163 (56.6) | 0.944 | 272 (55.4) | 204 (54.5) | 190 (59.0) | 0.456 |
| 3 years |  | 817 (74.9) | 854 (71.9) | 0.113 | 332 (78.1) | 297 (78.6) | 188 (65.3) | <0.001 | 366 (74.5) | 283 (75.7) | 205 (63.7) | 0.001 |
| Change | *P*^3^ | <0.001 | <0.001 | 0.673^4^ |  |  |  | 0.012^5^ |  |  |  | 0.001^5^ |
| 11. How many times per week do you consume commercial sweets or pastries (not homemade), such as cakes, cookies, biscuits, or custard? | <3 |  |  |  |  |  |  |  |  |  |  |  |
| Baseline |  | 754 (69.1) | 813 (68.5) | 0.750 | 307 (72.2) | 255 (67.5) | 192 (66.7) | 0.199 | 339 (69.0) | 257 (68.7) | 217 (67.4) | 0.199 |
| 3 years |  | 835 (76.5) | 865 (72.9) | 0.045 | 321 (75.5) | 301 (79.6) | 213 (74.0) | 0. 190 | 372 (75.8) | 265 (70.9) | 228 (70.8) | 0.170 |
| Change | *P*^3^ | <0.001 | 0.011 | 0.363^4^ |  |  |  | 0.271^5^ |  |  |  | 0.076^5^ |
| 12. How many servings of nuts (including peanuts) do you consume per week? (1 serving 30 g) | ≥3 |  |  |  |  |  |  |  |  |  |  |  |
| Baseline |  | 371 (34.0) | 416 (35.0) | 0.602 | 133 (31.3) | 147 (38.9) | 91 (31.6) | 0.046 | 160 (32.6) | 155 (41.4) | 101 (31.4) | 0.007 |
| 3 years |  | 601 (55.1) | 623 (52.5) | 0.213 | 181 (42.6) | 362 (95.8) | 58 (20.1) | <0.001 | 188 (38.3) | 353 (94.4) | 82 (25.5) | <0.001 |
| Change | *P*^3^ | <0.001 | <0.001 | 0.159^4^ |  |  |  | <0.001^5^ |  |  |  | <0.001^5^ |
| 13. Do you preferentially consume chicken, turkey, or rabbit meat instead of veal, pork, hamburger, or sausage? | Yes |  |  |  |  |  |  |  |  |  |  |  |
| Baseline |  | 694 (63.6) | 836 (70.4) | 0.001 | 265 (62.4) | 242 (64.0) | 187 (64.9) | 0.765 | 354 (72.1) | 255 (68.2) | 227 (70.5) | 0.457 |
| 3 years |  | 867 (79.5) | 1010 (85.1) | <0.001 | 350 (82.4) | 304 (80.4) | 213 (74.0) | 0.021 | 403 (82.1) | 332 (88.8) | 275 (85.4) | 0.023 |
| Change | *P*^3^ | <0.001 | <0.001 | 0.001^4^ |  |  |  | 0.177^5^ |  |  |  | 0.038^5^ |
| 14. How many times per week do you consume vegetables, pasta, rice, or other dishes seasoned with sofrito (sauce made with tomato and onion, leek, or garlic and simmered with olive oil)? | ≥2 |  |  |  |  |  |  |  |  |  |  |  |
| Baseline |  | 714 (65.4) | 745 (62.8) | 0.183 | 271 (63.8) | 249 (65.9) | 194 (67.4) | 0.598 | 313 (63.7) | 239 (63.9) | 193 (59.9) | 0.470 |
| 3 years |  | 847 (77.6) | 927 (78.1) | 0.791 | 371 (87.3) | 301 (79.6) | 175 (60.8) | <0.001 | 423 (86.2) | 306 (81.8) | 198 (61.5) | <0.001 |
| Change | *P*^3^ | <0.001 | <0.001 | 0.276^4^ |  |  |  | <0.001^5^ |  |  |  | <0.001^5^ |

^1^ P by χ2-test between Age groups. ^2^ P by χ2-test between Diet groups. ^3^ P by McNemar test between Baseline and 3 years. ^4^ P by χ2-test of changes between Age groups. ^5^ P by χ2-test of changes between Diet groups. ^*^ ≥1 portion raw or as a salad. Q1, ≤62 years old; Q4 ≥71 years old; tbsp, table spoon; EVOO, extra virgin olive oil; MedDiet+EVOO, Mediterranean diet supplemented with extra virgin olive oil; MedDiet+Nuts, Mediterranean diet supplemented with nuts.

**Supplementary Table 2. Changes in cardiovascular risk factors after 3 years of nutritional nutrition stratified by extreme quartiles of age.**

|  | **≤62 years old (Q1)** | | | | **≥71 years (Q4)** | | | |  |  |  |
| --- | --- | --- | --- | --- | --- | --- | --- | --- | --- | --- | --- |
|  | All  N= 1091 | MedDiet-EVOO  N= 425 | MedDiet-nuts  N= 378 | Control diet  N= 288 | All  N= 1187 | MedDiet-EVOO  N= 491 | MedDiet-nuts  N= 374 | Control diet  N= 322 | *P group* | *P age* | *P interac-tion* |
| Systolic blood pressure, mmHg | |  |  |  |  |  |  |  |  |  |  |
| Baseline | 146.1 (145 to 147.2) | 145.7 (144 to 147) | 147.7 (146 to 150) | 145 (143 to 147) | 152.7 (151.6 to 153.8) | 150.7 (149 to 152)^a^ | 152.5 (151 to 154)^ab^ | 155 (153 to 157)^b^ | 0.073 | <0.001 | 0.017 |
| Change 3 years | -4.6 (-5.6 to -3.6) | -3.6 (-5.1 to -2.03) ^a^ | -5.8 (-7.4 to -4.1)^b^ | -4.6 (-6.5 to -2.6)^a^ | -2.4 (-3.4 to -1.5) | -1.1 (-2.6 to 0.29)^a^ | -3.2 (-4.9 to -1.6)^b^ | -2.9 (-4.7 to -1.1)^a^ | 0.026 | 0.002 | 0.874 |
| Diastolic blood pressure, mmHg | |  |  |  |  |  |  |  |  |  |  |
| Baseline | +85.5 (84.9 to 86.1) | +85.3 (84.4 to 86.2) | +86.4 (85.4 to 87.3) | +84.9 (83.8 to 86) | +81.3 (80.7 to 81.8) | +80.9 (80 to 81.8) | +81.6 (80.6 to 82.5) | 81.3 (80.3 to 82.4) | 0.139 | <0.001 | 0.495 |
| Change 3 years | +1.68 (1 to 2.4) | +0.82 (-0.3 to 1.9) | +1.86 (0.7 to 3) | +2.37 (1.1 to 3.7) | -1.38 (-2 to -0.7) | -0.34 (-1.3 to 0.7) | -1.7 (-2.8 to -0.6) | -2.1 (-3.3 to -0.9) | 0.967 | <0.001 | 0.010 |
| Total Cholesterol, mg/dL | |  |  |  |  |  |  |  |  |  |  |
| Baseline | 210.1 (208 to 213) | 213.2 (210 to 217) | 210.3 (206 to 214) | 206.7 (202 to 211) | 205.9 (204 to 208) | 206.5 (203 to 210) | 207.5 (204 to 211) | 203.6 (199 to 208) | 0.069 | 0.010 | 0.515 |
| Change 3 years | -9.8 (-12.2 to -7.4) | -9.1 (-12.7 to -5.5) | -7.4 (-11.4 to -3.4) | -12.9 (-17.5 to -8.3) | -12.2 (-14.3 to -10) | -9.5 (-12.7 to -6.4) | -12.3 (-16 to -8.5) | -14.8 (-19 to -10.6) | 0.062 | 0.144 | 0.495 |
| LDL-Cholesterol, mg/dL | |  |  |  |  |  |  |  |  |  |  |
| Baseline | 132.7 (128 to 137) | 135.2 (128 to 142) | 128.1 (120 to 136) | 134.7 (127 to 142) | 131.7 (128 to 136) | 133.2 (127 to 139) | 133.8 (127 to 140) | 128.2 (121 to 136) | 0.608 | 0.753 | 0.265 |
| Change 3 years | -7.5 (-9.7 to -5.4) | -6.5 (-9.9 to -3.2) | -4.7 (-8.4 to -1) | -11.3 (-15.5 to -7.2) | -10.8 (-12.8 to -8.8) | -9.5 (-12.4 to-6.5) | -10.2 (-13.7 to -6.7) | -12.7 (-16.4 to -8.9) | 0.038 | 0.030 | 0.545 |
| HDL-Cholesterol, mg/dL | |  |  |  |  |  |  |  |  |  |  |
| Baseline | 50 (49.2 to 50.8) | 50.1 (48.9 to 51.3) | 49.7 (48.4 to 50.9) | 50.2 (48.7 to 51.7) | 51.9 (51.1 to 52.6) | 52.3 (51.2 to 53.4) | 51.5 (50.2 to 52.7) | 51.8 (50.4 to 53.2) | 0.573 | 0.001 | 0.896 |
| Change 3 years | -1.74 (-2.4 to -1.1) | -1.99 (-2.96 to -1) | -1.9 (-2.97 to -0.83) | -1.33 (-2.6 to -0.1) | -0.7 (-1.3 to -0.13) | -0.34 (-1.2 to 0.5) | -0.69 (-1.7 to -0.3) | -1.07 (-2.2 to 0.04) | 0.966 | 0.017 | 0.425 |
| Triglycerides, mg/dL |  |  |  |  |  |  |  |  |  |  |  |
| Baseline | 137.7 (133 to 143) | 139.6 (131 to 148) | 136.3 (128 to 145) | 137.3 (127 to 147) | 131 (126 to 136) | 128.9 (122 to 136) | 136.1 (128 to 145) | 128.1 (119 to 137) | 0.737 | 0.063 | 0.414 |
| Change 3 years | -5.86 (-10.1 to -1.6) | -5.56 (-12.1 to 1) | -5.25 (-12.5 to 2) | -6.76 (-15 to 1.5) | -5.79 (-9.6 to -1.9) | -6.36 (-12 to -0.68) | -5.6 (-12.4 to 1.2) | -5.42 (-12.9 to 2) | 0.981 | 0.983 | 0.955 |
| Glucose, mg/dL |  |  |  |  |  |  |  |  |  |  |  |
| Baseline | 121.7 (119 to 124) | 122.2 (118 to 126) | 119.5 (115 to 124) | 123.5 (118 to 129) | 121.5 (119 to 124) | 123.9 (120 to 128) | 120.9 (116 to 125) | 119.6 (115 to 124) | 0.426 | 0.881 | 0.417 |
| Change 3 years | -0.07 (-2.4 to 2.3) | +1.41 (-2.2 to 5) | -0.21 (-4.2 to 3.8) | -1.4 (-6 to 3.2) | -1.25 (-3.4 to 0.9) | -2.95 (-6.1 to 0.18) | -2.22 (-5.95 to 1.5) | +1.4 (-2.7 to 5.5) | 0.843 | 0.465 | 0.198 |
| Weight, Kg |  |  |  |  |  |  |  |  |  |  |  |
| Baseline | 80.2 (79.5 to 80.9) | 80.3 (79.2 to 81.4) | 80.7 (79.5 to 81.9) | 79.7 (78.3 to 81.1) | 73.6 (72.9 to 74.3) | 74.1 (73 to 75.1) | 73.8 (72.6 to 75) | 72.9 (71.6 to 74.2) | 0.276 | <0.001 | 0.834 |
| Change 3 years | -0.18 (-0.46 to 0.1) | -0.15(-0.59 to 0.29)^ab^ | +0.23 (-0.24 to 0.7)^a^ | -0.61(-1.14 to -0.08)^b^ | -0.81 (-1.08 to -0.54) | -1.08(-1.5 to -0.67)^ab^ | -0.47(-0.9 to 0.002)^a^ | -0.89 (-1.39 to -0.38)^b^ | 0.024 | 0.002 | 0.402 |
| BMI, Kg/m^2^ |  |  |  |  |  |  |  |  |  |  |  |
| Baseline | 30.1 (29.9 to 30.4) | 30.2 (29.9 to 30.6) | 30.2 (29.8 to 30.6) | 30 (29.5 to 30.4) | 29.7 (29.5 to 30) | 29.9 (29.6 to 30.2) | 29.6 (29.2 to 30) | 29.7 (29.3 to 30.1) | 0.466 | 0.015 | 0.742 |
| Change 3 years | -0.12 (-0.22 to -0.01) | -0.09 (-0.26 to 0.08) | +0.03 (-0.15 to 021) | -0.28 (-0.49 to -0.08) | -0.27 (-0.38 to -0.17) | -0.39 (-0.55 to -0.23) | -0.14 (-0.32 to 0.04) | -0.29 (-0.48 to -0.09) | 0.036 | 0.040 | 0.297 |
| Waist, cm |  |  |  |  |  |  |  |  |  |  |  |
| Baseline | 101 (100 to 102) | 101 (99.9 to 102) | 101 (100 to 102) | 101 (99.6 to 102) | 100 (99.5 to 101) | 101 (99.6 to 101) | 100 (99.1 to 101) | 99.6 (98.5 to 101) | 0.553 | 0.044 | 0.659 |
| Change 3 years | +0.07 (-0.33 to 0.45) | -0.02 (-0.6 to 0.6) | +0.44 (-0.2 to 1.1) | -0.23 (-0.99 to 0.53) | -0.12 (-0.49 to 0.26) | -0.01 (-0.58 to 0.56) | -0.14 (-0.81 to 0.52) | -0.19 (-0.90 to 0.52) | 0.601 | 0.519 | 0.582 |

Values are expresed as mean (95% CI). The analysis of the effect of the treatment group and the Quartile of Age (Q1 vs Q4) were performed by ANOVA for the analysis of the baseline visit and by ANCOVA adjusted with the baseline values for the change at 3 years. ^abc^ Treatment groups with different superscript letters show statistical differences according to the Bonferroni correction; simple effect contrasts were performed in the variables with significant "Group-Quartile of age" interaction. EVOO, extra virgin olive oil; MedDiet+EVOO, Mediterranean diet supplemented with extra virgin olive oil; MedDiet+Nuts, Mediterranean diet supplemented with nuts.
